# Detection of Quantitative Trait Loci Associated with Alkaline Tolerance Using Recombinant Inbred Line Population Derived from Longdao5 × Zhongyouzao8 at Seedling Stage

**DOI:** 10.3390/life14091151

**Published:** 2024-09-11

**Authors:** Xijuan Zhang, Kai Liu, Chuanming Yang, Benfu Hou, Xianli Yang, Lizhi Wang, Shize Cui, Yongcai Lai, Zhugang Li, Shukun Jiang

**Affiliations:** 1 Crop Cultivation and Tillage Institute of Heilongjiang Academy of Agricultural Sciences, Harbin 150086, China; xijuanzhang@haas.cn (X.Z.); mosangbikezaizheli@haas.cn (C.Y.); cc1@haas.cn (B.H.); yangxianli@haas.cn (X.Y.); lizhiwang@haas.cn (L.W.); xiaocui@haas.cn (S.C.); 2 Northeast Branch of National Salt-Alkali Tolerant Rice Technology Innovation Center, Harbin 150086, China; liukai@haas.cn (K.L.); laowantong@haas.cn (Y.L.); 3 Heilongjiang Provincial Key Laboratory of Crop Physiology and Ecology in Cold Region, Harbin 150086, China; 4 Heilongjiang Provincial Engineering Technology Research Center of Crop Cold Damage, Harbin 150086, China; 5 Qiqihar Branch of Heilongjiang Academy of Agricultural Sciences, Qiqihar 161006, China

**Keywords:** *japonica* rice, alkali tolerant, seedling stage, Northeast China, QTL

## Abstract

Salt–alkaline stress is one of the most stressful occurrences, causing negative effects on plant development and agricultural yield. Identifying and utilizing genes that affect alkaline tolerance is an excellent approach to accelerate breeding processes and meet the needs for remediating saline–alkaline soil. Here, we employed a mapping population of 176 recombinant inbred lines (RILs) produced from a cross between alkali-tolerant Longdao5 and alkali-sensitive Zhongyouzao8 to identify the quantitative trait loci (QTLs) determining alkali tolerance at the seedling stage. For the evaluation of alkali tolerance, the recovered seedling’s average alkali tolerance index (ATI), root number (RN), root length (RL), seedling dry weight (SW), root dry weight (RW), and seedling height (SH) were assessed, together with their relative alkaline damage rate. Under alkaline stress, the ATI was substantially negative connected with the root number, seedling height, seedling dry weight, and root dry weight; however, it was considerably positive correlated with the relative alkaline damage rate of the root number and root dry weight. A total of 13 QTLs for the root number, root length, seedling height, seedling dry weight, root dry weight, and alkali tolerance index under alkaline stress were identified, which were distributed across chromosomes 1, 2, 3, 4, 5, 7, and 8. All of these QTLs formed two QTL clusters for alkali tolerance on chromosome 5 and chromosome 7, designated *AT5* and *AT7*, respectively. Nine QTLs were identified for the relative alkaline damage rate of the root number, root length, seedling height, seedling dry weight, and root dry weight under alkali stress. These QTLs were located on chromosome 2, 4, 6, 7, 8, 9, and 12. In conclusion, these findings further strengthen our knowledge about rice’s genetic mechanisms for alkaline tolerance. This research offers clues to accelerate breeding programs for new alkaline-tolerance rice varieties.

## 1. Introduction

Soil saline-alkalization is one of the most serious abiotic stresses and is considered an important environmental limiting factor affecting global agroecosystems and food security [1]. It has numerous negative consequences for crops, including osmotic pressure, high pH stress, ionic balance disruption, limited nutrient uptake, and inhibition of plant growth, development, and yield [2,3]. According to the Food and Agriculture Organization (FAO), the total area of saline–alkaline land worldwide currently exceeds 1 billion hectares [4]. It is estimated that by 2050, more than 50% of arable land will be salinized due to factors such as low rainfall, high surface evaporation, irrational irrigation, poor agricultural practices, excessive use of chemical fertilizers, and other similar causes [5,6]. China is the third largest saline–alkaline soil country in the world, with a total saline–alkaline land area of 9.91 × 10^7^ hectares, which is mainly distributed in Northwest China, North China, Northeast China, and coastal areas [1,7]. The Songnen Plain in Northeast China is the largest distribution area of alkali-soda soils in the world, where the carbonates are the main composition of salts. The soil pH in this area is above 8.5 and reaches 9.0 to 10.5 in the highly alkaline soils [8]. A major problem in this area is alkali damage caused by high amounts of sodium carbonate (Na_2_CO_3_) or sodium bicarbonate (NaHCO_3_). Research and practice have shown that planting rice in saline–alkaline soil is one of the best ways to improve saline–alkaline soil and improve farmers ‘ income, agricultural benefit, and ecological environment [9]. With the advancement in saline–alkaline-tolerant crop breeding, such as rice, the mild (salinity: ≤0.3%, pH 7.1–8.5) and moderate (salinity: 0.3–0.6%, pH 8.5–9.5) saline–alkaline soils are expected to be reclaimed for crop production [5]. Compared with neutral saline soil, alkaline soil is more harmful to rice because it is not only subject to osmotic stress, ionic toxicity, and oxidative stress, but also has high pH, which destroys the integrity of cell membranes and root vitality and reduces photosynthesis function and ultimately affects plant growth [10,11]. Therefore, understanding the mechanisms of plant response to alkali stress and creating novel alkali-tolerant germplasm resources are necessary for the effective utilization of arable land and improving crop yields to meet the needs of world population growth [12,13].

Most previous studies have shown that alkaline tolerance is a typical polygenic systemic trait in rice. Many QTLs conferring alkaline tolerance in rice have been identified in the past decade using the linkage map of molecular markers and various genetic research populations [14,15,16,17,18,19,20,21]. Thirteen QTLs associated with the dead leaf rate (DLR) and six QTLs associated with the dead seedling rate (DSR) in the different rice growing periods after transplantation under alkaline stress were identified on demonstrated chromosomes 2, 3, 4, 6, 7, and 8. Chromosomes 9, 10, and 11 using an F_2:3_ population were derived from a cross between two *japonica* rice varieties, Gaochan106 and Changbai9 [14]. A total of 26 QTLs associated with alkaline tolerance at the early seedling stage were detected on chromosomes 1, 5, 6, 7, 8, 9, and 11 in the same F_2:3_ population, consisting of four QTLs for the root number, five QTLs for the relative alkaline damage rate of the root number, six QTLs for the root length, two QTLs for the relative alkaline damage rate of the root length, five QTLs for the seedling height, and four QTLs for the relative alkaline damage rate of seedling height [15]. Seven QTLs associated with germination under alkaline stress were also detected on chromosome 2, 5, 6, 7, 9, 10, and 11 using the same F_2:3_ population [16]. A total of seven QTLs for seedling day survival (SDS) and the concentrations of Na^+^ and K^+^ in shoots (SNC and SKC) and roots (RNC and RKC) under alkaline stress using an F_2:3_ population were derived from a cross between Caidao and WD20342. Of these QTLs, *qSNC3* was further narrowed down to an 81.7 kb region [17]. A total of eight QTLs associated with the score of alkalinity tolerance (SAT), shoot Na^+^ and K^+^ concentrations (SNC and SKC), and shoot Na^+^/K^+^ ratio (SNK) were identified through genome-wide association mapping in a population of 295 *japonica* rice varieties [18]. Eight QTLs and nine lead SNPs associated with alkaline tolerance at the bud stage were identified on chromosomes 1, 4, 5, 6, 7, 11, and 12 by using an RIL population (184 lines derived from a cross between Kongyu131 and Xiaobaijingzi) and a population of 295 *japonica* rice varieties [19]. The *qAT11* was also further narrowed down to a 218 kb region in this research [19]. Forty-two QTL intervals for 11 traits under alkaline stress were identified in 193 recombinant inbred lines (RILs) developed from a cross between Cocodrie and N22 [20]. Ninety QTLs and eight genes were identified as significantly associated with alkaline tolerance in a population of 428 diverse rice accessions [21].

Although the above studies provide a better understanding of the genetic basis of alkaline tolerance in rice, only a few genes associated with alkaline tolerance have been identified so far, including *ALT1*, *OsY3IP1*, *OsBBX17*, *GS3*, *OsSAP6*, *OsLOL5*, *OsSDG721*, and *OsDMI3* [4,22,23,24,25,26,27,28]. *ALT1* encodes an Snf2 family chromatin remodeling ATPase and negatively regulates alkaline tolerance through enhanced defense against oxidative stress in rice [22]. *OsY3IP1* encodes a nuclear-encoded thylakoid protein and increases alkaline stress tolerance by reducing reactive oxygen species accumulation in rice [23]. *OsBBX17* encodes a B-box transcription factor and regulates saline–alkaline tolerance via the MAPK cascade pathway in rice [24]. *GS3* encodes an atypical G protein g subunit that affects the phosphorylation of aquaporins to modulate the distribution of hydrogen peroxide (H_2_O_2_) and negatively regulate alkaline tolerance [4]. *OsSAP6* encodes a rice stress-associated protein and interacts with OsPK5 to positively regulate soda-salt–alkaline tolerance through ROS homeostasis in rice [25]. *OsLOL5* contains two LSD1-like-type C_2_C_2_ domains and improves alkaline tolerance via the active oxygen detoxification pathway [26]. *OsSDG721* positively regulates the expression level of high-affinity K^+^ transporter1;5 (OsHKT1;5) [27]. Furthermore, a calcium/calmodulin-dependent protein kinase, OsDMI3, was found to improve saline–alkaline tolerance by regulating Na^+^ and H^+^ influx in rice roots [28]. To date, the mechanisms of rice sensation and adaptation to high salt stress have been extensively studied [29,30]. However, the mechanisms of rice response to the high pH environment caused by alkaline stress remain largely unknown. The objectives of this research were to identify QTLs conferring alkaline tolerance from high-alkali-tolerant *japonica* rice “Longdao5” using an RIL population derived from the cross between Longdao5 and middle-alkaline-resistant *indica* rice variety “Zhongyouzao8”.

## 2. Materials and Methods

### 2.1. Plant Materials

A high-alkaline-tolerant *japonica* rice variety, ‘Longdao5,’ bred by our research group, was licensed for release in 2006 and is widely known for its exceptional tolerance to alkaline conditions. It has become extensively cultivated in the sodic soil of Heilongjiang Province in Northeast China. To investigate the genetic basis of alkali tolerance in ‘Longdao5’, we meticulously selected ‘Zhongyouzao8’, which was bred by China National Rice Institute as a medium-alkaline-resistant high-yield *indica* rice variety, to construct a mapping population. A recombinant inbred line (RIL) mapping population including 176 F_9_ lines was generated by the single-seed descents method from a cross between Longdao5 as the female parent and Zhongyouzao8 as the male parent. The RILs and their parents were grown using conventional cultivation methods at the experimental farm of the Heilongjiang Academy of Agricultural Sciences in 2023.

### 2.2. Linkage Map Construction

The genetic map was constructed using IciMapping software version 4.2. The ‘MAP’ function was used to divide the markers into 12 chromosomes with a LOD of 4.0 and check the final marker order [31]. The linkage map was re-sorted again by R/qtl software version 1.66 to obtain the final linkage map [32]. Finally, a linkage map of the RIL population was constructed with 213 markers, including 155 simple sequence repeat (SSR) loci, 40 sequence-tagged site (STS) loci, and 18 insertion and deletion (InDel) loci. This linkage map covered a total length of 1420.05 cM, with an average distance of 6.67 cM between adjacent markers [33]. The linkage map was also drawn using R/qtl software version 1.66 [32].

### 2.3. Alkaline-Tolerance Evaluation

All experimental seeds of the parents and RIL population were braked with dormancy at 50 °C for 48 h, then surface sterilized with 1% HgCl_2_ for 10 min and germinated in the dark at 28 °C. Seedlings were grown in Kimura’s culture solution B [22] for 4 weeks until they reached 2.5 leaves and then transferred to the same culture solution supplemented with or without 80 mM NaHCO_3_ (pH = 8.8) for 12 days. After alkaline stress treatment, rice seedlings were washed twice with deionized water and then transferred to the Kimura’s culture solution B for recovery for 6 days. These solutions were renewed every 3 days. Ten plants were randomly selected from each treatment after 6 days of recovery, and the root number (RN), root length (RL), seedling dry weight (SW), root dry weight (RW), and seedling height (SH) were measured. Three independent replicates were set up, and the mean value of the three replicates was used for the QTL analysis.

**Figure 1:**
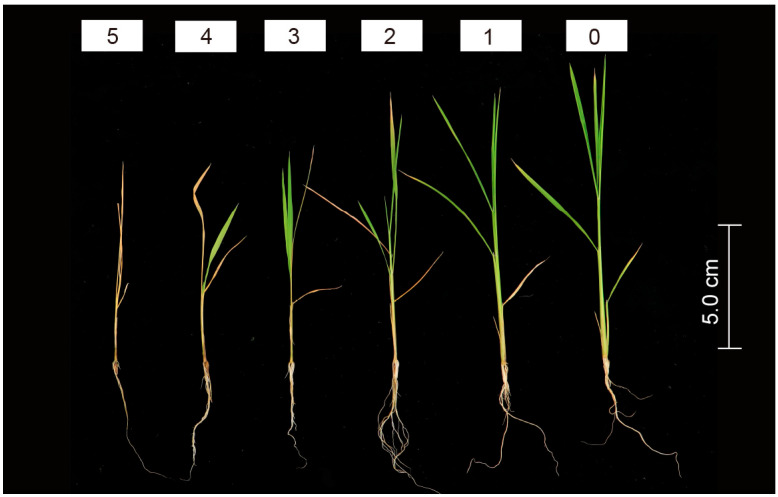
The evaluation criteria for the relative degree of leaf alkaline damage of rice seedlings.

A six-class rating scale by measuring the average alkali tolerance index (ATI) value of the recovered seedling was used to evaluate the relative degree of leaf damage of seedlings. Class 5: all seedlings were dead; Class 4: most leaves were withered and yellow with center leaves green; Class 3: 30 percent of leaves were greyish and yellow; Class 2: 50 percent of leaves were greyish and yellow; Class 1: seedling growth was slightly suppressed in appearance, and only the leaf tip shriveled; Class 0: no damage, and all leaves were normal in color (Figure 1) [34].

The relative alkaline damage rates for the root number, root length, root dry weight, seedling dry weight, and seedling height were calculated according to the following formula: Relative alkaline damage rate (%) = (control value − alkaline treatment value)/control value × 100 [15].

### 2.4. QTL Mapping for Alkali Tolerance

The arithmetic mean values of all the above traits and index from three replicates were used to evaluate the alkaline tolerance for each line. The QTL analysis was performed by using the inclusive composite interval mapping (ICIM) method of QTL IciMapping software version 4.2 (http://www.isbreeding.net, accessed on 1 January 2020) [31]. The LOD significance thresholds for QTL significance were determined by 1000 permutations at *p* < 0.05, and the walking speed was 1.0 cM.

## 3. Results

### 3.1. Seedling Performance between Longdao5 and Zhongyouzao8 under Alkaline Stress

**Figure 2:**
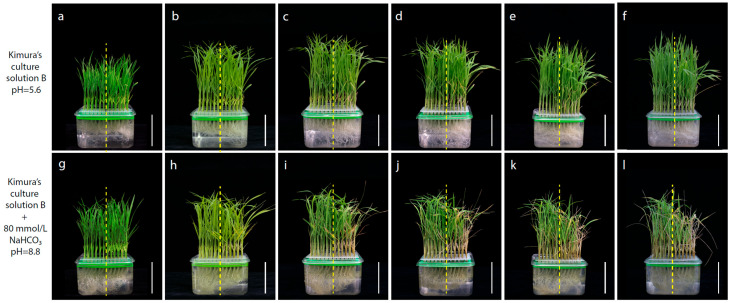
The phenotypic traits associated with alkaline tolerance at the seedling stage between Longdao5 (left) and Zhongyouzao8 (right); (**a**) 0 days in normal Kimura’s culture solution B; (**b**) 6 days in normal Kimura’s culture solution B; (**c**) 9 days in normal Kimura’s culture solution B; (**d**) 12 days in normal Kimura’s culture solution B; (**e**) 15 days in normal Kimura’s culture solution B; (**f**) 18 days in normal Kimura’s culture solution B; (**g**) 0 days in Kimura’s culture solution B with NaHCO_3_; (**h**) 6 days in Kimura’s culture solution B with NaHCO_3_; (**i**) 9 days in Kimura’s culture solution B with NaHCO_3_; (**j**) 12 days in Kimura’s culture solution B with NaHCO_3_; (**k**) 3 days of recovery in Kimura’s culture solution B; (**l**) 6 days of recovery in Kimura’s culture solution B. The bars = 5.0 cm.

The phenotypic traits related to alkaline tolerance at the seeding stage between Longdao5 and Zhongyouzao8 were identified by using Kimura’s culture solution B with (pH 5.6) or without 80 mM NaHCO_3_ (pH 8.8) (Figure 2). On the sixth day of alkaline stress treatment, it can be seen that the second complete leaf of the lower part of Zhongyouzao8 began to fade and yellow, while Longdao5 did not change accordingly (Figure 2b,h). On the 9th and 12th day of alkaline stress treatment, most of the leaves of Zhongyouzao8 began to wilt, while only the second leaf of Longdao5 showed wilting (Figure 2c,d,i,j). After recovery, the new leaves of Longdao5 recovered quickly and the seedling height grew obviously, while the recovery of Zhongyouzao8 was slow, and the seedling height hardly changed within 6 days of recovery (Figure 2e,f,k,l).

**Figure 3:**
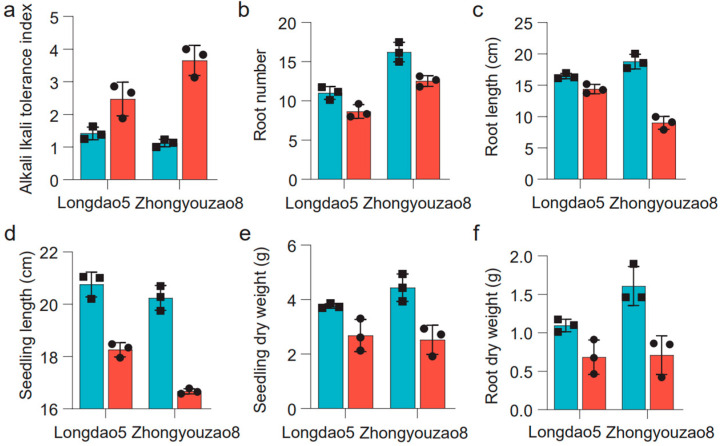
The phenotypic traits associated with alkaline tolerance between Longdao5 and Zhongyouzao8 after 6 days of recovery in Kimura’s culture solution B. CK: cyan; Alkaline stress: carmine pink. (**a**) The alkali tolerance index between Longdao5 and Zhongyouzao8; (**b**) the root number between Longdao5 and Zhongyouzao8; (**c**) the root length between Longdao5 and Zhongyouzao8; (**d**) the seedling height between Longdao5 and Zhongyouzao8; (**e**) the seedling dry weight between Longdao5 and Zhongyouzao8; (**f**) the root dry weight between Longdao5 and Zhongyouzao8.

There were significant differences in the alkaline tolerance index, root number, root length, root dry weight, seedling dry weight, and seedling height between Longdao5 and Zhongyouzao8 when exposed to 80 mM NaHCO_3_ (Figure 3). Under alkaline stress, the alkaline tolerance index of the alkali-tolerant parent Longdao5 (2.47 ± 0.52) was obviously lower than that of the medium-alkali-resistant parent Zhongyouzao8 (3.65 ± 0.46) (Figure 3a, Table 1). The root number of Longdao5 decreased from 3.76 ± 0.09 to 2.68 ± 0.59, while the root number of Zhongyouzao8 decreased from 4.34 ± 0.51 to 2.52 ± 0.53 (Figure 3b, Table 1). The root length of Longdao5 decreased from 16.45 ± 0.44 cm to 14.38 ± 0.75 cm, while the root length of Zhongyouzao8 decreased from 18.77 ± 1.17 cm to 8.99 ± 1.03 cm (Figure 3c, Table 1). The seedling height of Longdao5 decreased from 20.76 ± 0.47 cm to 18.26 ± 0.27 cm, while the seedling height of Zhongyouzao8 decreased from 20.24 ± 0.48 cm to 16.67 ± 0.10 cm (Figure 3d, Table 1). The seedling dry weight of Longdao5 decreased from 3.76 ± 0.09 g to 2.68 ± 0.59 g, while the seedling dry weight of Zhongyouzao8 decreased from 4.44 ± 0.51 g to 2.52 ± 0.53 g (Figure 3e, Table 1). The root dry weight of Longdao5 decreased from 1.10 ± 0.08 g to 0.69 ± 0.22 g, while the root length of Zhongyouzao8 decreased from 1.61 ± 0.25 g to 0.71 ± 0.25 g (Figure 3f, Table 1). In a word, Longdao5 has stronger alkali resistance than Zhongyouzao8.

### 3.2. Phenotypic Variation in the RIL Population under Alkaline Stress

**Figure 4:**
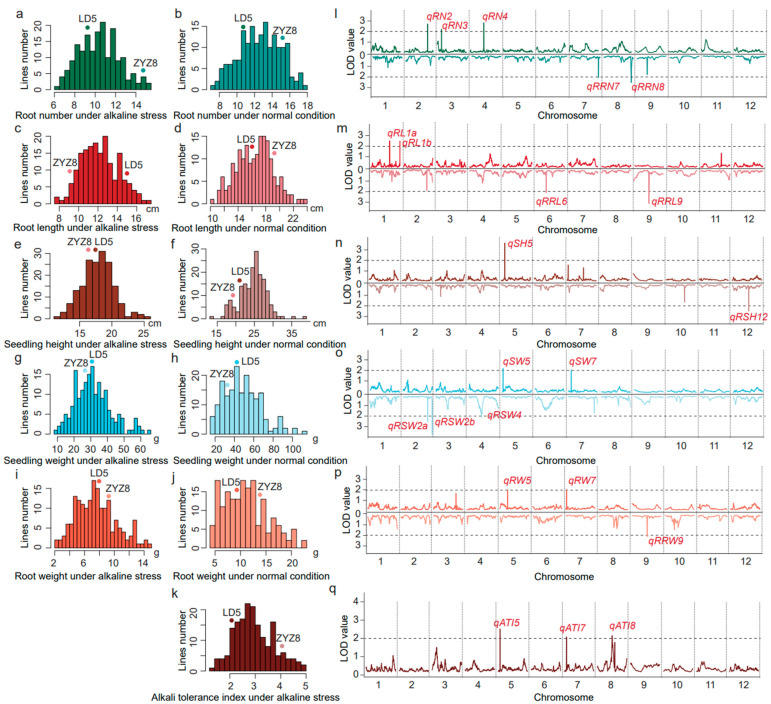
Distributions and the LOD value for the root number (RN), root length (RL), seedling height (SH), seedling weight (SW), root weight (RW), and alkali tolerance index (ATI) under the alkaline stress and normal condition at the whole-genome level in RIL populations. (**a**) The root number distribution of RILs’ population under alkaline stress; (**b**) the root number distribution of RILs’ population under the normal condition; (**c**) the root length distribution of RILs’ population under alkaline stress; (**d**) the root length distribution of RILs’ population under the normal condition; (**e**) the seedling height distribution of RILs’ population under alkaline stress; (**f**) the seedling height distribution of RILs’ population under the normal condition; (**g**) the seedling weight distribution of RILs’ population under alkaline stress; (**h**) the seedling weight distribution of RILs’ population under the normal condition; (**i**) the root weight distribution of RILs’ population under alkaline stress; (**j**) the root weight distribution of RILs’ population under the normal condition; (**k**) the alkali tolerance index distribution of RILs’ population under alkaline stress; (**l**) the LOD value of the root number (the upper part) and its relative alkaline damage rate (the lower part) at the whole-genome level in RILs’ populations; (**m**) the LOD value of root length (the upper part) and its relative alkaline damage rate (the lower part) at the whole-genome level in RILs’ populations; (**n**) the LOD value of seedling height (the upper part) and its relative alkaline damage rate (the lower part) at the whole-genome level in RILs’ populations; (**o**) the LOD value of seedling weight (the upper part) and its relative alkaline damage rate (the lower part) at the whole-genome level in RILs’ populations; (**p**) the LOD value of root weight (the upper part) and its relative alkaline damage rate (the lower part) at the whole-genome level in RILs’ populations; (**q**) the LOD value of the alkali tolerance index at the whole-genome level in RILs’ populations.

The means, standard deviations, and range of the alkali tolerance index, root number, root length, root dry weight, seedling dry weight, and shoot height at the seedling stage of RILs are shown in Table 1. The root number in the RIL population varied from 0.08 to 46.83 under normal conditions and from 6.00 to 15.19 under alkaline stress (Table 1). The root length in the RIL population varied from 0.27 cm to 52.91 cm under normal conditions and from 7.83 cm to 17.08 cm under alkaline stress (Table 1). The seedling height in the RIL population varied from 4.96 cm to 47.55 cm under normal conditions and from 11.19 cm to 25.41 cm under alkaline stress (Table 1). The seedling dry weight in the RIL population varied from 2.41 g to 77.31 g under normal conditions and from 9.67 g to 64.88 g under alkaline stress (Table 1). The root dry weight in the RIL population varied from 0.62 g to 76.21 g under normal conditions and from 2.44 g to 14.77 g under alkaline stress (Table 1). The alkali tolerance index in the RIL population varied from 1.36 to 5.00 under alkali stress (Table 1). The frequency distribution of the alkali tolerance index, root number, root length, root dry weight, seedling dry weight, and seedling height all showed almost normal continuous distribution under control and alkali stress conditions (Figure 4a–k, Table 1). All the above traits exhibited significant transgressive segregations with trait values either larger or smaller than those of Longdao5 or Zhongyouzao8 (Figure 4a–k, Table 1). This information indicated that all of the above traits were quantitative traits controlled by multiple genes and were suitable for the QTL analysis.

### 3.3. The Correlation Coefficients between the ATI and the Seedling Traits in the RIL Population

**Figure 5:**
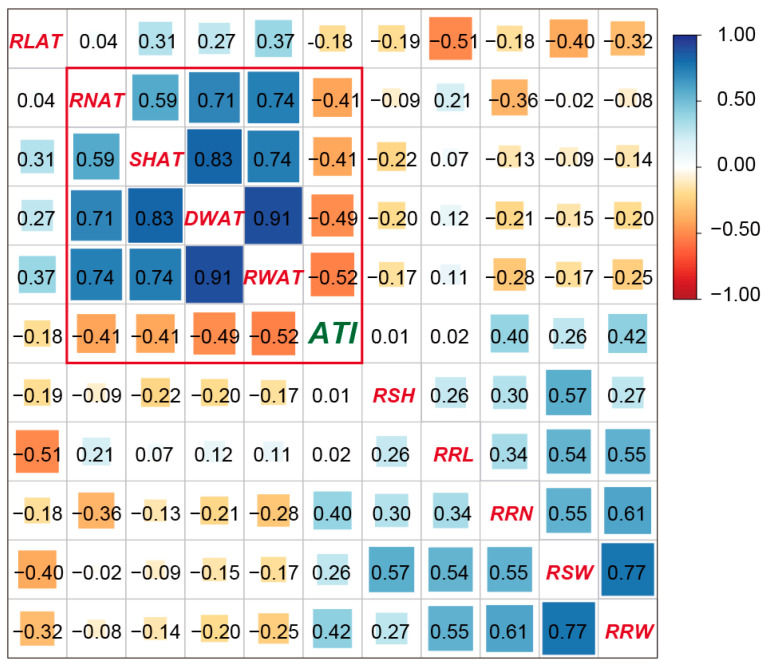
The correlation coefficients between the ATI and the seedling traits in the RIL population. The values are correlation coefficients. The areas of squares correspond to absolute values of the corresponding r. The blue and orange colors indicate positive and negative correlations, respectively.

The correlation coefficients between the ATI and the ten seedling and root traits under alkaline stress in the RIL population are shown in Figure 5. The ATI was significantly negatively correlated with the root number, seedling height, seedling dry weight, and root dry weight under alkaline stress, while the ATI was significantly positively correlated with the relative alkaline damage rate of the root number and root dry weight. This information indicated that the root number, seedling height, seedling dry weight, root dry weight, and relative alkaline damage rate of the root number and root dry weight were important for alkali tolerance in rice.

### 3.4. QTL Mapping for Seedling- and Root-Related Traits under Alkali Stresses

A total of 13 QTLs were identified for the root number, root length, seedling height, seedling dry weight, root dry weight, and alkali tolerance index under alkali stress. These QTLs were distributed over chromosome 1, 2, 3, 4, 5, 7, and 8. The explanation for the phenotypic variation by a single QTL varied from 6.23% to 10.73% (Figure 5, Table 2). Three QTLs (*qATI5*, *qATI7*, and *qATI8*) for the alkali tolerance index were detected on chromosome 5, 7, and 8 with the LOD score of 2.60, 2.11, and 2.19, respectively (Figure 4q). Additive alleles of *qATI5* and *qATI8* originated from Longdao5, but additive alleles of *qATI7* originated from Zhongyouzao8 (Table 2). Three QTLs (*qRN2*, *qRN3*, and *qRN4*) for the root number under alkali stress were detected on chromosome 2, 3, and 4 with the LOD score of 2.77, 2.17, and 2.82, respectively (Figure 4l). Additive alleles of *qRN2* and *qRN4* originated from Zhongyouzao8, while additive alleles of *qRN3* originated from Longdao5 (Table 2). Only one QTL-*qSH5* associated with seedling height under alkali stress was identified on chromosome 5 with a LOD value of 3.55 and the biggest phenotypic variation of 10.73% (Figure 4n). The additive alleles of *qSH5* originated from Longdao5 with an additive effect of 0.68 (Table 2). Two QTLs, *qRL1a* and *qRL1b*, that controlled root length under alkali stress were identified on chromosome 1 (Figure 4m). Their additive alleles all originated from Longdao5 with the LOD score of 2.51 and 2.52, respectively (Table 2). The *qSW5* and *qSW7* for seedling dry weight under alkali stress were mapped on chromosome 5 and 7 (Figure 4o). They explained 7.06% and 6.23% of the total phenotypic variance, respectively. Additive alleles of *qSW5* originated from Longdao5, while additive alleles of *qSW7* originated from Zhongyouzao8 (Table 2). Two QTLs for root dry weight under alkali stress were identified on chromosome 5 and 7 and named *qRW5* and *qRW7* (Figure 4p). The *qRW5* and *qRW7* explained 6.23% and 6.32% of the total phenotypic variance, respectively. Additive alleles of *qRW5* also originated from Longdao5, while additive alleles of *qRW7* also originated from Zhongyouzao8 (Table 2). All these QTLs formed two QTL clusters for alkali tolerance on chromosome 5 and chromosome 7 named *AT5* and *AT7*, respectively.

**Figure 6:**
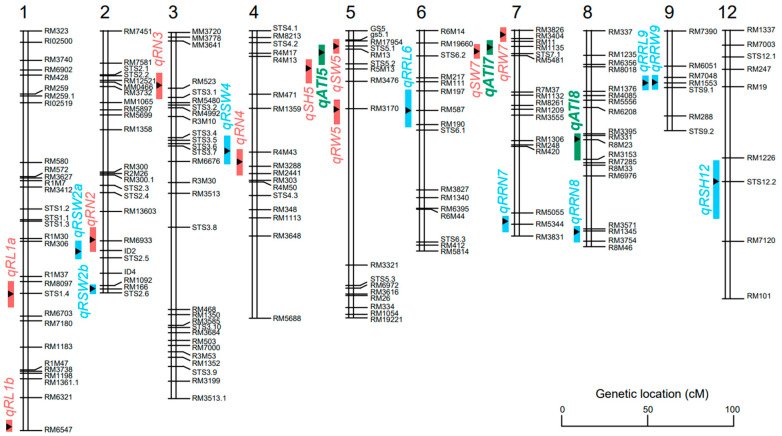
Genomic locations of the QTLs with alkaline tolerance-related traits identified in the RIL population. Green indicates the QTLs for the key trait alkali tolerance index; carmine pink indicates the QTLs for the traits of a seedling and root under alkali stress; blue indicates the QTLs for the relative alkaline damage rate; 1–12 indicates chromosome 1 to chromosome 12.

A total of nine QTLs were identified for the relative alkaline damage rate of the root number, root length, seedling height, seedling dry weight, and root dry weight under alkali stress. These QTLs were distributed on chromosome 2, 4, 6, 7, 8, 9, and 12. The explanation for the phenotypic variation by a single QTL varied from 6.23% to 10.96%. The LOD value of each single QTL varied from 2.01 to 3.63 (Figure 6, Table 3). Two QTLs (*qRRN7* and *qRNN8*) for the relative alkaline damage rate of the root number were detected on chromosome 7 and 8 with the LOD score of 2.04 and 2.54, respectively (Figure 4l). Additive alleles of *qRRN7* originated from Longdao5, but additive alleles of *qRNN8* originated from Zhongyouzao8 (Table 3). Two QTLs (*qRRL6* and *qRRL9*) for the relative alkaline damage rate of root length were detected on chromosome 6 and 9 with the LOD score of 2.13 and 3.17, respectively (Figure 4m). Additive alleles of *qRRL9* originated from Longdao5, but additive alleles of *qRRL6* originated from Zhongyouzao8 (Table 3). Only one QTL-*qRSH12* associated with the relative alkaline damage rate of seedling height under alkali stress was identified on chromosome 12 with a LOD value of 2.49 and a phenotypic variation of 7.65% (Figure 4n, Table 3). Three QTLs (*qRSW2a, qRSW2b,* and *qRSW4*) for the relative alkaline damage rate of seedling dry weight under alkali stress were identified on chromosome 2 and 4 (Figure 4o). They explained 7.36%, 10.96%, and 6.23% of the total phenotypic variance, respectively. Additive alleles of *qRSW2b* originated from Longdao5, while additive alleles of *qRSW2a* and *qRSW4* all originated from Zhongyouzao8 (Table 3). One QTL for the relative alkaline damage rate of root dry weight under alkali stress was identified on chromosome 9 and named *qRRW9* (Figure 4p). The *qRRW9* explained 6.29% of the total phenotypic variance. And its additive allele also originated from Longdao5 (Table 3).

## 4. Discussion

In contrast to salt stress caused by neutral salts (e.g., NaCl and Na_2_SO_4_), alkaline stress caused by basic salts such as NaHCO_3_ and Na_2_CO_3_ has a completely different genetic mechanism [5]. Numerous studies have shown that alkaline stress is a complex trait controlled by multiple quantitative genes (QTLs) in rice [14,15,17,19]. A total of 13 QTLs were identified for the root number, root length, seedling height, seedling dry weight, root dry weight, and alkali tolerance index under alkali stress on chromosome 1, 2, 3, 4, 5, 7, and 8 in this research. Five QTL alleles had positive effects inherited from the alkali-tolerant parent Longdao5, while the remaining QTL alleles each had positive effects inherited from the alkali-tolerant parent Zhongyouzao8. This indicates that the alkaline tolerance genes are not entirely derived from the alkaline-tolerant parents. Some alkaline tolerance genes may also be hidden in the alkaline-sensitive parents and expressed through hybridization and genetic recombination. Xiao et al. detected two high-yield QTLs that increased yield by 18% and 17%, respectively, from low-yielding Malaysian wild rice [35]. More than half of the QTLs related to cold tolerance at the seedling stage and tillering stage detected by Han et al. [36] came from the cold-sensitive parent. The other researchers also found the presence of “hidden genetic variation” for improved cold tolerance in apparently cold-susceptible indica rice donors in cold-tolerance research [37]. This similar phenomenon often occurs in abiotic stress breeding and genetic research of rice, such as cold tolerance, heat tolerance, and salt and alkali tolerance. However, the genetic mechanism is still unclear and requires further investigation.

The comparison of the locations between our identified QTLs with previously reported QTLs indicated that *qRN2*, *qRN4*, *qRW5*, *qRW7*, *qSW7*, *qATI7*, *qRSW2a*, *qRSW2b*, *qRSW4*, *qRRL6*, *qRRN8*, *qRRL9*, and *qRRW9* were detected as major QTLs in previous research [14,15,16,17,19]. For example, *qRN2* controlling the root number under alkaline stress, *qRSW2a* controlling relative seedling dry weight under alkaline stress, and *qRSW2b* controlling relative seedling dry weight under alkaline stress were identified in the same region with *qDLR2-1* for the dead leaf rate under alkaline stress [14]. *qRW5* for root dry weight under alkaline stress was detected in the same region with *qRSH5* for the relative alkaline damage rate for seedling height [15]. The saline–alkaline-tolerant gene *OsDMI3* encoding calcium/calmodulin-dependent protein kinase was also found in this region [28]. *qRW7*, *qSW7*, and *qATI7*, controlling the root dry weight, seedling dry weight, and alkali tolerance index under alkaline stress, were identified in the same region with *qDLR7-1* for the dead leaf rate under alkaline stress [14] and *qRGC7* for the germination rate under alkaline stress [16]. *qRSW4* for controlling relative seedling dry weight under alkaline stress and *qRN4* controlling the root number under alkaline stress were identified in the same region with *qDLR4* for the dead leaf rate under alkaline stress [14]. *qRRL6* for controlling relative root length under alkaline stress was re-identified in Qi et al.’s report [19]. *qRRN8* controlling the relative root number under alkaline stress was identified in the same region with *qSNC8* for the concentrations of Na^+^ in shoots under alkaline stress [17]. *qRRL9* controlling relative root length and *qRRW9* controlling relative root dry weight under alkaline stress were identified in the same region with *qRL9* for root length under alkaline stress [15]. Two cloned genes, *ALT1* and *OsY3IP1*, were found in the *qRL1a* locus [22,23]. The predicted ALT1 protein belonged to the Ris1 subgroup of the Snf2 family and negatively influenced alkaline tolerance mainly by defending against oxidative damage. It represents a possible two-step strategy to improve the tolerance of rice plants to alkaline stress [22]. The OsY3IP1 protein shared significant homology with various Y3IP1 family members. The increased tolerance conferred by *OsY3IP1* overexpression correlated with reduced reactive oxygen species accumulation [23]. Among our identified QTLs, only *qRL1a*, *qRL1b*, *qRN3*, *qSH5*, *qSW5*, *qATI5*, *qRRN7*, *qATI8*, and *qRSH12* were identified for the first time.

We focused on the QTL cluster on chromosome 5 (*AT5*). The *AT5* locus was mapped in a 2.2 Mb region with 197 genes between RM17954 (3,651,365 bp) and R5M13 (5,992,698 bp) according to the rice annotation project database (http://rapdb.dna.affrc.go.jp/). The next step is to narrow down the *AT5* interval and screen candidate genes by constructing a larger mapping population combined with a transcriptome analysis.

Similar to studies on drought, cold, heat, and other abiotic stresses, accurate evaluations of the saline–alkaline–tolerant phenotype of rice plants are a decisive factor in mapping saline–alkali-tolerant QTL. The average alkali tolerance index (ATI) value of the recovered seedling was successfully used to evaluate the relative degree of leaf damage of seedlings in this study. An evaluation index similar to ATI has also been widely used in the cold-tolerance evaluation of rice seedlings, cold-tolerance evaluation of rice buds, and salt- and alkali-tolerance evaluation of maize [34,38]. In this study, ATI was found to be significantly negatively correlated with the root number, seedling height, seedling dry weight, and root dry weight under alkaline stress (Figure 5). No strong correlation was found between relative alkaline damage rates for the root number, root length, root dry weight, seedling dry weight and seedling height, and ATI. Therefore, in the study of saline–alkali-tolerance gene mapping in rice, the traits that directly reflect the growth state of the plant should be used instead of the relative damage reduction value.

Developing saline–alkaline-tolerant crops is the most fundamental and efficient strategy to counteract soil salinization and ensure food security worldwide. In recent decades, the rapid development in molecular genetics and functional genomics has made it possible to excavate favorable alleles for saline–alkali tolerance and to introduce or transfer them into elite varieties [10,24,29,39]. Molecular breeding combined QTL mapping with marker-assisted selection and accelerates and more accurately identifies genes related to abiotic stress tolerance, which has been successfully applied for enhancing abiotic stress of elite rice varieties. For example, submergence tolerance conferred by *Sub1A* has been expeditiously introgressed by marker-assisted breeding into popular high-yielding variety IR64 that is grown in flood-prone regions of Asia [39]. *Saltol*, a favorable QTL controlling rice shoot Na^+^/K^+^ homeostasis under salt stress from variety Pokkali, was successfully introgressed into commercial varieties through marker-assisted breeding [39]. Over the past few decades, super high yield and better quality and resistance to multiple diseases have been the main goals of rice breeding in Northeast China, resulting in the lack of salt and alkaline tolerance of most modern rice cultivars in this region [13]. In this study, we identified five QTL alleles that had positive effects inherited from the alkali-tolerant parent Longdao5. These favorable QTLs would solve the problem of limited progress in developing salt-tolerant *japonica* rice varieties due to the lack of both high-salt-tolerance genetic resources and reliable salt-tolerance genes.

## 5. Conclusions

We identified 13 QTLs for the root number, root length, seedling height, seedling dry weight, root dry weight, and alkali tolerance index under alkaline stress and 9 QTLs for the relative alkaline damage rate. In addition, these QTLs formed two QTL clusters for alkali tolerance on chromosome 5 and chromosome 7 named *AT5* and *AT7*, respectively. The additive alleles of *AT5* originated from alkali-tolerant variety Longdao5, while the additive alleles of *AT7* originated from alkali-sensitive variety Zhongyouzao8. These two QTL clusters should be targeted for introgression to improve alkalinity tolerance in rice.

## Figures and Tables

**Table 1:** The phenotypic traits related to alkaline tolerance of Longdao5, Zhongyouzao8, and their RIL population.

Traits	Parents		RIL Population				
	Longdao5	Zhongyouzao8	Mean ± SD	Range	CV (%)	Skewness	Kurtosis
RNAT	2.68 ± 0.59	2.52 ± 0.53	10.43 ± 1.96	6.00–15.19	18.81	0.22	−0.41
RN	3.76 ± 0.09	4.34 ± 0.51	16.58 ± 10.67	0.08–46.83	64.34	0.40	−0.57
RLAT	14.38 ± 0.75	8.99 ± 1.03	12.20 ± 1.90	7.83–17.08	15.55	0.21	−0.51
RL	16.45 ± 0.44	18.77 ± 1.17	24.62 ± 13.11	0.27–52.91	53.24	−0.07	−0.97
SHAT	18.26 ± 0.27	16.67 ± 0.10	17.82 ± 2.45	11.19–25.41	13.74	0.18	0.31
SH	20.76 ± 0.47	20.24 ± 0.48	27.17 ± 7.07	4.96–47.55	26.03	−0.12	0.36
SWAT	2.68 ± 0.59	2.52 ± 0.53	31.39 ± 10.91	9.67–64.88	34.76	0.62	0.26
SW	3.76 ± 0.09	4.44 ± 0.51	32.24 ± 16.23	2.41–77.31	50.36	0.32	−0.34
RWAT	0.69 ± 0.22	0.71 ± 0.25	7.62 ± 2.68	2.44–14.77	35.19	0.47	−0.25
RW	1.10 ± 0.08	1.61 ± 0.25	28.03 ± 18.48	0.62–76.21	65.92	0.27	−0.91
ATI	2.47 ± 0.52	3.65 ± 0.46	2.96 ± 0.76	1.36–5.00	25.82	0.40	−0.27

RNAT: Root number under alkali stress; RN: Root number in normal condition; RLAT: Root length under alkali stress; RL: Root length in normal condition; SHAT: Seedling height under alkali stress; SH: Seedling height in normal condition; SWAT: Seedling dry weight under alkali stress; SW: Seedling dry weight in normal condition; RWAT: Root weight under alkali stress; RW: Root weight in normal condition; ATI: alkali tolerance index.

**Table 2:** Alkali-resistant QTLs identified in LD5-ZYZ8 RILs.

QTL	Trait	Chr. ^a^	Peak Marker	QTL Interval	LOD ^b^	Var (%) ^c^	Add. ^d^	Positive Allele
*qATI5*	Alkali tolerance index	5	RM13	STS5.1-STS5.2	2.60	7.98	−0.15	LD5
*qATI7*	Alkali tolerance index	7	RM1135	RM11-STS7.1	2.11	6.53	0.12	ZYZ8
*qATI8*	Alkali tolerance index	8	R8M23	RM331-RM3153	2.19	6.76	−0.06	LD5
*qRN2*	Root number	2	RM6933	RM13603-ID2	2.71	8.30	0.43	ZYZ8
*qRN3*	Root number	3	RM523	MM3641-STS3.1	2.17	6.70	−0.31	LD5
*qRN4*	Root number	4	RM3288	R4M43-RM2441	2.82	8.62	0.44	ZYZ8
*qSH5*	Seedling height	5	R5M13	STS5.2-RM3476	3.55	10.73	−0.68	LD5
*qRL1a*	Root length	1	STS1.4	RM8097-RM6703	2.51	7.71	−0.48	LD5
*qRL1b*	Root length	1	RM6547	RM6321-RM6547	2.52	7.74	−0.42	LD5
*qSW5*	Seedling dry weight	5	RM17954	gs5.1-RM13	2.29	7.06	−2.74	LD5
*qSW7*	Seedling dry weight	7	STS7.1	RM1135-RM5481	2.01	6.23	2.07	ZYZ8
*qRW5*	Root dry weight	5	RM3170	RM3476-RM3321	2.01	6.23	−0.54	LD5
*qRW7*	Root dry weight	7	RM3404	RM3826-RM11	2.04	6.32	0.61	ZYZ8

^(a)^ Chromosome; ^(b)^ Logarithm (base 10) of odds for corresponding QTL peak; ^(c)^ Percentage of explained phenotypic variation by corresponding QTL; ^(d)^ Additive effect of corresponding QTL.

**Table 3:** The relative alkaline damage rate QTLs identified in LD5-ZYZ8 RILs.

QTL	Trait	Chr. ^a^	Peak Marker	QTL Interval	LOD ^b^	Var (%) ^c^	Add. ^d^	Positive Allele
*qRRN7*	Relative root number	7	RM5344	RM5055-RM3831	2.04	6.32	−1.95	LD5
*qRRN8*	Relative root number	8	RM1345	RM3571-RM3754	2.54	7.80	0.04	ZYZ8
*qRSH12*	Relative seedling height	12	STS12.2	RM1226-RM7120	2.49	7.65	−1.52	LD5
*qRRL6*	Relative root length	6	RM587	RM197-RM190	2.13	6.58	3.57	ZYZ8
*qRRL9*	Relative root length	9	RM1553	RM7048-STS9.1	3.17	9.64	−3.32	LD5
*qRSW2a*	Relative seedling dry weight	2	ID2	RM6933-STS2.5	2.39	7.36	2.12	ZYZ8
*qRSW2b*	Relative seedling dry weight	2	RM166	RM1092-STS2.6	3.63	10.96	−3.47	LD5
*qRSW4*	Relative seedling dry weight	4	R4M43	RM1359-RM3288	2.01	6.23	3.16	ZYZ8
*qRRW9*	Relative root dry weight	9	RM1553	RM7048-STS9.1	2.03	6.29	−3.57	LD5

^(a)^ Chromosome; ^(b)^ Logarithm (base 10) of odds for corresponding QTL peak; ^(c)^ Percentage of explained phenotypic variation by corresponding QTL; ^(d)^ Additive effect of corresponding QTL.

## Data Availability

The raw data supporting the conclusions of this article will be made available by the authors on request.
